# Are Andrija Štampar’s principles gone – forever and ever?

**DOI:** 10.3325/cmj.2017.58.372

**Published:** 2017-10

**Authors:** Aleksandar Džakula, Rafaela Tripalo, Dorja Vočanec, Dagmar Radin, Karmen Lončarek

**Affiliations:** 1 Andrija Štampar School of Public Health, University of Zagreb, Zagreb, Croatia *rafaela.tripalo@gmail.com*; 2 Faculty of Political Science, University of Zagreb, Zagreb, Croatia; 3 School of Medicine, University of Rijeka, Rijeka, Croatia

The work, results, and thoughts of Andrija Štampar are recognized globally as a public health heritage (1). They encompass the vision of health as a state of overall well-being, society as the key responsible health care provider, and a set of values and guiding principles underlining the importance of optimal health. The reasons behind their adoption worldwide lie in the success of public health projects in Croatia (former Yugoslavia) in 1920s promoted by Zagreb School of Public Health (SPH) (later SPH A. Štampar), established in 1927. The key mission of the SPH was to develop further the public health practice and promote Štampar’s public health principles (2).

Andrija Štampar’s principles (Box 1) advocate universal and widely understood values in different social contexts. Apart from their widely understood meaning, the principles also serve as a guideline for policy makers and governments to promote a just and fair society, equality, and equity (3). Whereas these principles illustrate the general determinants of health system organization in some societies, in Croatia they have been acclaimed and accepted in general as a comprehensive and best public health practice. In some way, we have kept the inherited Štampar principles and practice as a professional “dogma” and unquestionable public health business model.

Box 1 **Dr Andrija Štampar’s principles**

**Table:** 

**Dr Andrija Štampar’s principles as a foundation for public health and socialized medicine (****3****)** *1. It is more important to enlighten the people than to impose the laws; therefore, the medical profession consists of only three short laws.* *2. It is most important to prepare the ground in a certain sphere and to develop the right understanding for questions of hygiene.* *3. The question of public health and its improvement must not be monopolized by medical authorities, but has to be cared for by everybody, for only by joint work can the progress of health be obtained.* *4. First of all, the physician must be a social worker; by individual therapy he cannot attain much, social therapy is the means of success.* *5. Economically the physician must not be dependent on his patient, because it hinders him in the accomplishment of his principal tasks.* *6. In matters of national health, no difference is to be made between the rich and the poor.* *7. It is necessary to form a health organization, in which the physician will seek the patient, not the patient the physician; for this is the only way to gather an ever-increasing number of those whose health we have to care for.* *8. The physician has to be the teacher of the people.* *9. The question of national health is of a greater economic than humanitarian importance.* *10. The principal fields of action of a physician are human settlements and not laboratories and consulting rooms.*

However, data from the World Health Organization (WHO) and the European Semester 2017 show that Croatia is lagging in some key public health indicators: health outcomes are below the European Union (EU) average and point to a challenge in preventing non-communicable chronic diseases (4,5). These public health data have come as an unpleasant surprise to the public health system in Croatia, given the 90 years of strong and pioneering public health practices based on Štampar’s globally recognized and acclaimed principles.

Prompted by the unfavorable statistics, we decided to explore how the current understanding of Štampar’s principles has evolved over time and whether the mismatch between the principles and outcomes has resulted from the principles themselves, our understanding of these principles, or some other reason. We analyzed their practical use over the last 90 years, ie, from the establishment of the SPH until today. We also examined the extent to which the principles are appropriate today to be taken into consideration in public health discussions, using a system of keywords, public health information, and experience in public health programs and interventions from 1920 until today.

## The Andrija Štampar’s principles

Štampar’s principles were presented publicly in 1926 through the examples of public health interventions performed since 1920 in the former Kingdom of Serbs, Croats, and Slovenes. Štampar’s formulation of the principles was based on the common values of a just society – equity and solidarity. His focus was on the context and problems facing the population and on the use of the existing public health methods and health technologies. As a visionary and revolutionary, Štampar tried to find innovative ways to fight infectious diseases and tackle burning socio-medical problems, such as the social determinants of health. It was important to reach out to the most disadvantaged groups, especially in the rural areas, which were the most populated areas at the time. Social conditions included poverty, poor living conditions, and hygiene-related problems. Generally, there was a perceivable lack of resources for public health interventions. However, the possibilities for a better coverage of the population and greater engagement of the existing resources were limited by the general lack of education, knowledge, and access to information. Health literacy was low. Since any contact with health authorities relied mostly on the individual physician-patient relationship, Štampar recognized the need to build a complete public health system. This system was going to be not only an addition to the state apparatus, but also a new technology that would transfer new knowledge into practice. Additionally, its purpose was to direct public health interventions. Using his political position and professional authority, Štampar demonstrated the power of policy technology.

## The principles – first reception and influence

Andrija Štampar implemented his principles and demonstrated their power in action in the poor and rural societies of the former Kingdom of Serbs, Croats, and Slovenes (1920-1929), and China (1933-1936). In part, they were recognized as a “model adjusted to the rural population”. However, the texts he had written during his trip to the United States (US) in 1931 and again in 1938 expand on the interpretation of his principles and advised the readers to think outside the box. During his stay in the US, Štampar recognized the problems facing an industrialized nation, particularly in urban areas affecting the newly vulnerable population – the unemployed workers and their families. Coming from the underdeveloped country, which was not a representative and universal setting of the time, Štampar encouraged a certain distance from the context in which the principles were created in order to make them universally applicable (3).

His observations that originated during his work in the US were not necessarily incorporated in his presentation and interpretation of his work. Namely, Štampar actively encouraged health care providers to adapt his principles to their own social environment and refer to them as guidelines only.

Moreover, during his stay in the US, Štampar recognized the role of different novelties: stakeholders, technologies, and the expectations in medicine and public health significantly changed. Altogether, he witnessed the new driving forces, which led to the modern health care development (6).

## Global perspectives and ideologies

Following significant public health achievements of the 1920s and 1930s, the rise of the Nazi state in Germany, and gross violations of human rights and horrific crimes of World War II, there was a focus shift away from public health topics and toward those of survival. Consequently, a new movement aimed at re-establishing the well-being of humankind characterized the post-war period. This new movement created a new definition of health and resulted in the establishment of the WHO. Moreover, the United Nations Assembly adopted the Declaration on Human Rights in response to the outrageous violations of human rights and threats to life and health. Štampar was one of the founding fathers of the WHO, which underlines his significance as a key player in the universal health care discourse.

The post-World War II period was marked by global economic growth, significant improvements in living conditions, and new advances in technology and medicine. The 1960s were recognized as the most intense time of growth and development, including many social movements and new ideas. Forty years after Štampar’s principles had been written, Yugoslavia experienced its biggest surge in socialism. Štampar’s principles became a living practice, and were considered one of the foundations of the Yugoslav socialist society. Thus, they ceased to exist as an active strategy and turned into a new socialist “dogma”, promoting the strength and stability of the new society. Such stability (or at least, the illusion of it) was very important and was prioritized over individuals - the system was highly regulated and seemingly highly structured. Most health care indicators and trends were positive. This “honey moon” lasted until the end of the 1970s, when the negative economic trends were followed by a demographic transition and low efficiency in health care (7). The deep economic and political crisis during 1980s was a slow introduction into the fall of socialism, encouraging a complete re-evaluation of the principles and visions upon which the Croatian society had been built.

## From the transition period to the present

During the 20th century, Štampar’s principles were integrated into all international organizations forming the basis of global health standards. However, the new Croatia had mislabeled many of the Štampar’s values and achievements as socialist heritage and communist ideology. This egregious misinterpretation of his principles had serious negative consequences on Croatian health care for the following decade. Namely, the fall of socialism and the transition to democracy, aggravated by the Homeland War in Croatia, once again put the public and national health in the spotlight. While Štampar’s principles had been globally accepted as guidelines for building a just society, Croatia failed to reach its critical and analytical potential. During this third period of Štampar’s principles, when they were needed more than ever, Croatia failed to perceive them critically. After the transition, Štampar’s approach was recognized as socialist and obsolete professional “nagging” and as such, it has been neglected, underestimated, and even ridiculed.

Instead of critically analyzing Štampar’s principles as guidelines within the today’s context, including equality, equity, and empowerment, we have replaced it by ideologically driven fear, actively avoiding a critical perspective on principles. This has led to a situation in which there remain only two alternatives: either to take Štampar’s principles literally and apply them without contextualization or to dismiss them completely as a relic of socialism.

## Content and context

In order to analyze these principles, it is necessary to deconstruct the context in which they emerged and became accepted and to recognize their fundamental public health intentions and contents. Moreover, it is important to identify health care technologies that relate them to the present-day context.

There are three key time points (the 1920s, the 1960s, and the present) in which Croatia, to its detriment, demonstrated a lack of critical approach toward Štampar’s principles. The social environment of this period was promoting non-critical thinking, along with disapproval of any questioning. Consequently, Štampar’s principles were perceived as unable to keep up with the development of society.

Specifically, in the 1920s, our region was faced with the burden of morbidity caused by infectious diseases, mostly among the rural population. The reasons for the easy and dramatic spreading of diseases were, among other factors, low health literacy and significant lack of awareness about the social determinants of health (3). Considering this, we may ask how the principles that emerged in that social context can be applied today.

First, if we substitute infectious diseases with chronic diseases associated with lifestyle habits, it is evident that both types of diseases are strongly associated with poverty and social deprivation. Additionally, if we replace the lack of information with information overload, which makes it difficult to distinguish the right messages from alternative realities, we find ourselves facing a new type of illiteracy. Also, if the lack of resources and knowledge about medicine from the 1920s is replaced with the recent explosive growth of knowledge and technology, we see that the world is facing a new challenge of “policy”: the challenge of how to turn knowledge into an effective intervention. Finally, we are left wondering how to prevent the process of commodification that is turning the patient-physician relationship from the one based on care, responsibility, and trust into one that is based solely on service and profit.

Over the last 30 years, the world community has strongly embraced Štampar’s principles, starting with the Ottawa Charter emphasizing empowerment and health literacy related to social determinants, and ending with the *Health 2020*, issued by the WHO, and focusing on inter-sector cooperation, justice, and equality, thus highlighting community management and policies (8,9).

Part of the answer to the question of why the implementation of Štampar’s principles faced permanent obstacles can be found in three long-lasting processes that go back to the time when Štampar worked. The first process involves the role and power of the state in securing the public interest, in this case public health. The second process refers to the diminished power of interventions driven by public institutions, including decreased trust in those institutions. Lastly, the third process includes the role and social position of health professions to achieve the public good. In the last 20 years, we have observed a significant weakening of state interventions toward socially deprived populations, resulting in a steady rise in social inequalities. On the other hand, during Štampar’s time, state interventions of the monarchy were the key mechanism for achieving public health priority goals (3). Along with the state’s weakening influence, increasing complexity of public services decreased the authority of health care institutions for the long-term changes, such as life style changes and health promotion and prevention (10). Consequently, the implementation of modern expertise and policy technologies that these institutions should implement has failed repeatedly. This process is global and affects a broad scope of public activities, including development of professionalism and society as a whole (11,12). Finally, the process of commodification is continually trying to push health care from the area of social welfare and onto the market, thus replacing care with services (13,14). This process had already been present in Štampar’s time when there were open attacks on him and his work. After a period of stagnation, almost identical processes are witnessed today (15).

It seems that Štampar’s insight into role of the state and market in health care was not well understood in Croatia, despite the fact that during his lifetime his insights were respected and valued in the world community. For example, during his visit to the US, he took part in numerous discussions about the meaning and purpose of the public health care system and the implementation of health insurance models similar to the ones in the developed parts of Europe. In fact, even President Roosevelt emphasized the need for health insurance during the 1938 National Health Conference in Washington, attended by Andrija Štampar. Štampar noticed that even in wealthy countries, the issue of national health care was of prime importance.

The importance of health as the biggest national resource, and the responsibility of the state and society for health care provision are probably the biggest and most important takeaways from Štampar's heritage (6).

## Conclusions

Štampar’s principles are part of the global health heritage, which form the basis of the formation and development of modern national health system. In the present-day setting, Štampar’s principles can be deconstructed and described by the following key words: social determinants, public health, health illiteracy, equity, health technology, health policy, and health care system. All these concepts are of high importance for current health authorities and professionals.

However, if their application is to be successful, they should first be deconstructed to determine their significance and relation to the context. Štampar’s principles have shown persistence, timelessness, and global appeal, surviving different stages and conceptual transitions of health systems and health policies, from the Lalonde Report and Medical Nemesis by Ivan Illich, to the Black Report and the Ottawa Charter (9,16-18).

Burdened by the weight of the socialist remains and the slow pace of health care reforms, Croatia failed to comprehend the universal values of Štampar's work and principles, but retained the academic historical discourse about them. Given the lack of evaluation of the performance of the Croatian health care system, even the international success of Štampar’s work has not been perceived as relevant and achievable in Croatian health care. Increasing social inequalities, negative trends in health care and health system, identified by the relevant international institutions (ie, EU Semester, EU Index), indicate that Croatia must promptly devote its health care politics to better understanding and implementing of the main public health principles (4,5,19). Public health professionals need to respect the standards and values promoted by the western health care systems and work in accordance with best practices to ensure optimal outcomes, while taking full responsibility for their work. Only then will they fully comply with Štampar’s practice.
